# Rutting Performance Evaluation of BMD Surface Mixtures with Conventional and High RAP Contents under Full-Scale Accelerated Testing

**DOI:** 10.3390/ma16247611

**Published:** 2023-12-12

**Authors:** Bilin Tong, Jhony Habbouche, Gerardo W. Flintsch, Brian K. Diefenderfer

**Affiliations:** 1 Virginia Tech Transportation Institute, Blacksburg, VA 24061, USA; gflintsch@vtti.vt.edu; 2 Virginia Transportation Research Council, Charlottesville, VA 22903, USA; jhony.habbouche@vdot.virginia.gov (J.H.); brian.diefenderfer@vdot.virginia.gov (B.K.D.)

**Keywords:** high RAP, asphalt concrete, balanced mix design, rutting performance, aging, accelerated pavement testing, heavy vehicle simulator, correlations, specifications, thresholds

## Abstract

The balanced mix design (BMD) constitutes a significant step forward in the pursuit of better-performing asphalt mixtures. This approach/framework offers increased innovative opportunities for the proper design and production of engineered asphalt mixtures without the need to strictly adhere to traditional volumetric requirements. The primary objective of this paper is to conduct a comprehensive investigation of the permanent deformation (rutting) behavior of surface mixtures (SMs) with conventional and high reclaimed asphalt pavement (HRAP) contents through full-scale accelerated testing under incremental loading conditions while accounting for the environmental aging effect. HRAP SMs were designed in this study, marking the initial application of Virginia Department of Transportation (VDOT) BMD special provisions, with attempts to incorporate 45% and even 60% RAP. Results showed that all BMD HRAP mixtures exhibited higher rut depths compared to the control mixture, which can be attributed to the inclusion of high binder contents aimed at enhancing cracking resistance. The asphalt pavement analyzer (APA) rut test and the stress sweep rutting tests were performed on mixtures sampled during production. Correlation analysis revealed significant and strong positive correlations between accelerated pavement testing (APT) and the multilevel laboratory rutting performance tests considered in this study. Finally, while acknowledging the limitations and all the assumptions considered in this study, the correlation analysis recommended refining the VDOT BMD APA rut depth threshold by lowering the current limit of 8 mm to 7 mm to ensure good performing mixtures from a rutting point of view.

## 1. Introduction

The Virginia Department of Transportation (VDOT) has continuously supported efforts to improve mixture performance. In 1997, the Superpave system, which utilizes performance-graded (PG) binders, was adopted to address rutting in Marshall mixtures. Adoption continued as the VDOT began using Superpave to design mixtures in 2000, and full implementation occurred in 2002 [1]. However, it was quickly noticed that many of the early Superpave-designed mixtures were coarse and dry, resulting in shorter service lives than desired. This initiated an effort to improve service lives [1]. To increase binder contents, the VDOT changed the gyratory compaction effort from the American Association of State Highway and Transportation Officials (AASHTO)-specified traffic-dependent level to 65 gyrations. Additional research by Maupin [1] revealed that binder contents determined during the Superpave design had not significantly changed from those of the previous Marshall mixtures, although differences in mixture gradations may have influenced the results. As a result, the VDOT implemented further changes to mix design requirements, including reducing the design gyrations to 50, along with making gradation and volumetric adjustments [2]. These adjustments were aimed at refining the mixture characteristics and ensuring better mixture performance. In addition to the efforts to improve durability, the VDOT has been responding to increased interest in the use of higher reclaimed asphalt pavement (RAP) contents [3].

The use of RAP greatly influences asphalt mixture production and development worldwide. While RAP usage has increased throughout the years, its growth plateaued at around 20% in the United States (U.S.) in 2014 [4,5]. In the U.S., the environmental and economic benefits gained using RAP in asphalt mixtures have encouraged state agencies to introduce special provisions and specifications allowing its use at relatively higher contents in mixtures [6,7]. The primary concern with high RAP (HRAP) asphalt mixtures is that they can become overly stiff due to the increased amount of RAP used, making them more brittle and prone to premature cracking [7]. West et al. documented challenges arising from the use of HRAP asphalt mixtures, which can be effectively addressed by increasing the binder content or incorporating recycling agents (RAs) and/or softer binders within a performance-based framework, such as a Balanced Mix Design (BMD) approach [8,9]. The BMD method replaces some aspects of traditional volumetric design with performance testing criteria for the most common distresses, such as rutting and cracking [9]. Although BMD mixtures cannot compensate for an unsound underlying pavement structure or the selection of inappropriate maintenance treatments, the BMD constitutes a significant step forward in the pursuit of better-performing asphalt mixtures. This framework/approach provides an opportunity to properly design and produce engineered asphalt mixtures, including those with higher RAP contents.

Distresses in flexible pavements refer to various types of damage or deterioration that can occur over time due to traffic loads, environmental factors, and material deficiencies. These distresses can significantly impact the structural integrity and serviceability of the road surface. Rutting stands as a significant distress that adversely affects the durability and service life of flexible pavement structures. This form of deterioration not only diminishes user comfort and compromises safety but also leads to escalated operational expenses for highway maintenance and rehabilitation. Understanding the extent to which the rutting resistance of pavement mixtures can be optimized requires a thorough investigation. Given agencies’ concerns about the long-term performance of pavements, research on rutting investigation has become an essential and indispensable area of study. Addressing this issue will contribute to the development of more robust and longer-lasting flexible pavements, benefiting both road users and maintenance authorities.

### 1.1. Virginia BMD Efforts

State highway agencies, including the VDOT, have shown interest in implementing the BMD for designing and accepting asphalt mixtures. The VDOT’s motivation for exploring the BMD stemmed from concerns over increased surface cracking and the desire to incorporate innovative mixture designs. Specifically, the VDOT sought to characterize the performance of mixtures with HRAP and other additives, which could not be adequately accounted for using traditional volumetric design approaches [10]. In 2017, the VDOT initiated studies to incorporate performance requirements into mix design using the BMD method. Performance tests, including the Cantabro test, indirect tensile cracking test (IDT-CT), and asphalt pavement analyzer (APA) rut test, were selected to assess durability, resistance to cracking, and resistance to rutting, respectively [11]. Two VDOT BMD special provisions, one for conventional and one for HRAP surface mixtures (SMs), were then drafted and revised for use in pilot projects. Field trials were conducted in 2019, marking the first applications of these BMD specifications [10]. Further field trials were planned and conducted in 2020 and 2022 to evaluate mixtures with higher RAP contents, softer binders, RAs, and other additives such as fibers and softening oils [10].

### 1.2. Rutting Performance Evaluation

Laboratory performance tests play a crucial role in the BMD process as they ensure the production of high-performing materials. Over the past few decades, several testing methods and analysis protocols have been proposed and adopted in the laboratory to characterize the rutting resistance of asphalt mixtures. These performance tests include the APA [12], Hamburg wheel tracking [13], French simulator [14], and uniaxial repeated load test to determine the Flow Number [15]. However, these procedures typically employ pass-fail criteria based on fixed testing conditions, which limits their ability to capture the complex nature of permanent deformation under varying field conditions. For instance, as part of the VDOT BMD specifications, the APA rut test is conducted at 64 °C, with a threshold of a maximum rut depth of 8.0 mm after 8000 loading cycles. To address this limitation and achieve a more comprehensive characterization of resistance to permanent deformation, two testing protocols have emerged in recent years: the Triaxial Stress Sweep (TSS) and the Stress Sweep Rutting (SSR) tests. The TSS test offers advantages over existing models by considering the influence of temperature, loading time, and deviatoric stress on permanent deformation behavior [16]. Additionally, the SSR test has been introduced to further simplify the TSS test, aiming to reduce its complexity and cost while maintaining its effectiveness in evaluating the resistance to permanent deformation [16]. Furthermore, Meroni et al. proposed a classification of rutting performance tests into three levels—basic, intermediate, and advanced—based on various factors, including complexity, cost, time, and training requirements [17]. This classification offers flexibility in selecting the appropriate level of testing depending on the specific objectives and constraints of a given research or engineering project.

### 1.3. Background on Accelerated Pavement Testing

In addition to performance testing and evaluation in the laboratory, accelerated pavement testing (APT) serves as a valuable tool to bridge the significant gap between mechanistic-empirical (ME) models developed using laboratory material testing characterization and the actual long-term pavement performance monitoring. APT offers a means to assess the performance of full-scale constructed pavements in an accelerated manner. This involves deliberately applying wheel loading, typically surpassing the design load limit, to a pavement system to assess its response within a significantly condensed timeframe. Therefore, it provides insights that complement long-term pavement performance monitoring and analysis [18]. This accelerated evaluation is accomplished through several means, including intensifying the number of load repetitions, adjusting loading conditions, introducing specific climatic conditions in terms of temperature and moisture, implementing thinner pavements with reduced structural capacity, or employing a combination of these factors [19].

Many institutions around the world have established and used APT facilities to conduct research on various topics. These institutions were able to achieve significant outcomes. For example, in South Africa, the focus of development has included but is not limited to large aggregate mix bases, granular emulsion mixes, rehabilitation measures for cemented-base pavements, and porous asphalt [20]. In California, numerous pavement sections have undergone testing involving materials such as dense-graded asphalt concrete, asphalt rubber, aggregate base, cement-treated base, and asphalt-treated permeable base, and others [20]. In Finland and Sweden, the VTT and VTI research institutes have tested typical Finnish pavement structures and evaluated mill and fill maintenance treatments for pavements with progressively increasing bearing capacities [20]. The Florida Department of Transportation (FDOT) has conducted evaluations on the effects of polymer modification of Superpave mixtures, rutting performance of coarse and fine-grained mixtures, the implementation of PG 76-22 Asphalt Rubber Binder in Florida, and others [20,21]. Furthermore, rutting evaluation of hot and warm mix asphalt concrete under high aircraft tire pressure and temperature has been carried out at the National Airport Pavement and Material Research Center [22].

In the U.S., Virginia is among the states that have implemented the use of APT facilities for pavement evaluation purposes. In 2015, a collaborative experiment between the Virginia Transportation Research Council (VTRC), the VDOT, and Virginia Tech was initiated and executed at the Virginia APT facility located at the Virginia Tech Transportation Institute (VTTI). The experiment encompassed three major studies, including the investigation of the impact of different overlays on cold central plant recycling (CCPR) material [23], the assessment of field performance of two distinct dense-graded SMs designed with varying design gyration levels, and the examination of reflection cracking. In 2020, another effort was planned and initiated to assess the application of the BMD concept in designing durable and longer-lasting surface mixtures in Virginia, with a particular emphasis on mixtures incorporating relatively higher RAP contents. The overarching scope of the effort included the development of the APT experiment, verification of mix design performance, documentation of paving operations and construction practices, and mixture sampling during production. Moreover, it included coring of as-placed material, the testing and analysis of the volumetric and performance properties of mixtures, actual testing using a heavy vehicle simulator (HVS) for rutting and cracking studies, analyses of collected pavement responses and generated data, and documentation of observations and lessons learned. A total of six experimental-testing lanes were constructed. These lanes featured the use of typical and high RAP contents, RA, a softer binder, and a warm mix asphalt (WMA) additive.

## 2. Objective and Scope

The primary objective of this paper is to conduct a comprehensive investigation of the permanent deformation (rutting) behavior of SMs with conventional and high RAP contents through full-scale accelerated testing under incremental loading conditions and the impact of the BMD on the rutting performance of these mixtures. This paper specifically covers the design of pavement test cells, the traffic test plan, and the measurement of rutting performance. In addition, this paper proposes an empirical rutting model to account for the environmental aging effect observed in the APT measurements. Furthermore, correlation analysis is conducted between APT rut depth and two laboratory rutting performance tests. Finally, an attempt to validate and refine the current VDOT BMD threshold is performed.

## 3. Research Significance

The use of high RAP content has significantly increased in the base layer of asphalt pavement, especially with the application of cold recycling techniques, enabling the potential for a 100% utilization rate of RAP [23]. Nonetheless, concerns persist regarding the performance of high RAP contents when used in the exposed surface layer subjected to heavier traffic loads and environmental aging. To expand RAP usage while addressing risks associated with HRAP mixtures, adopting a performance-based mix design method, such as the BMD, proves crucial.

This study evaluated HRAP SMs, marking the initial application of the VDOT BMD special provisions. The aim was to incorporate 45% and even 60% RAP into the mixtures. Given the absence of long-term performance data for HRAP pavements, a comprehensive investigation of the designed mixtures was conducted in the laboratory and under full-scale testing. This involved bridging the gap between multiple-level laboratory performance tests and APT. This approach enabled the validation and refinement of current VDOT BMD thresholds, providing valuable insights for ongoing long-term performance monitoring efforts in the field.

## 4. APT Setup for Virginia 2020 BMD Experiment

Conventional laboratory test protocols primarily aim to simulate the stress and strain conditions experienced under typical traffic scenarios. However, it is important to acknowledge that the stress or strain conditions imposed on specimens in laboratory settings may differ from those encountered in the field. To bridge this gap between laboratory simulations and/or ME design models developed using laboratory material testing characterization and actual pavement stress conditions, APT tests can be conducted using representative truck wheel loads and tire pressures. Additionally, it provides a valuable approach to overcome the limitations inherent in conventional laboratory tests and offers a more precise representation of the stress conditions experienced by pavements in actual field conditions.

**Figure 1:**
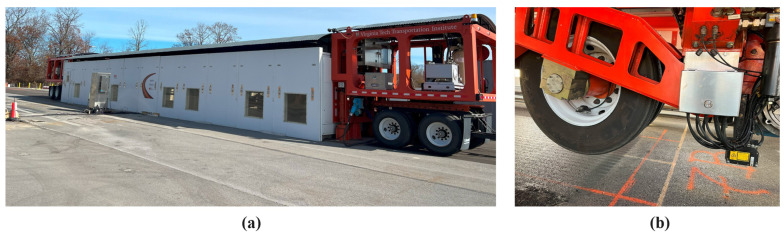
Photographs Taken of (**a**) Dynatest Mark VI Heavy Vehicle Simulator; (**b**) Laser Profiler Mounted on HVS Carriage.

The central component of the APT BMD experiment was the Dynatest HVS system (Dynatest Consulting Inc., Gainesville, FL, USA), specifically the Dynatest Mark VI model shown in Figure 1a. This system featured an environmental chamber designed to maintain a consistent temperature at the loaded area. The HVS system automatically regulates the surface temperature to ensure that a depth of 50.8 mm (2 inches) from the surface is maintained at the temperature of interest. Inside the environmental chamber, a dual tire carriage was mounted, capable of achieving a speed of up to 20 km/h (12.4 mph) with a tolerance of ±3.2 km/h (2 mph). The loading was applied using a dual tire assembly utilizing 11.00R22.5 tires inflated to a pressure of 105 psi. Additionally, the system allowed for the completion of 12,000 uni-directional passes or 24,000 bi-directional passes within a 24-h period. The dual tire carriage was capable of accommodating loads ranging from 30 kN to 100 kN (6750 lbf to 22,500 lbf). A laser profiler installed on the HVS carriage, as shown in Figure 1b, was used to conduct scans of the pavement surface and quantify the vertical permanent deformation present at the surface. A data acquisition system (DAS) was employed to capture signals from various instruments and monitor the conditions of the pavement. To facilitate this data acquisition process, input modules from National Instruments (Budapest, Hungary) were installed in a chassis along with a controller. The LabVIEW software (2019) was utilized to develop an interactive interface for data management and analysis. The DAS system, along with its associated components, was housed in a weather-proof chamber located near the HVS apparatus.

## 5. Experimental Program

### 5.1. Materials Evaluated

A total of one control and five BMD 9.5 mm dense-graded SMs were investigated in this study. The mixtures incorporated various combinations of RAP contents (determined based on the total weight of the mixtures), two binders (PG 64S-22 and PG 58-28), one RA, and one WMA additive (the same for all six mixtures). The primary function of the WMA additive was to act as a liquid antistrip. The mixtures were defined as follows:

- 30_C: a non-BMD mixture serving as a control (C) and including 30% RAP content and PG 64S-22 asphalt binder. This mixture was designed following the conventional Superpave mix design methodology, through which the optimum binder content (OBC) was selected for voids in the total mixture (VTM) of 4%.
- 30_O: a BMD-optimized version of mixture 30_C (with O denoting optimized) featuring the use of 30% RAP content and a PG 64S-22 at a relatively higher OBC when compared to mixture 30_C. The optimization process involved adjusting the aggregate gradation and increasing the asphalt binder content.
- 45_HR: a BMD-optimized HRAP mixture featuring the use of 45% RAP and a PG 64S-22. This mixture design resulted in a much higher OBC as compared with all other evaluated mixtures. No softer binder or RA was used during the design and production of this mixture.
- 45_HR_RA: a BMD-optimized HRAP mixture featuring the use of 45% RAP, a typical PG 64S-22, and an RA.
- 45_HR_L: a BMD-optimized HRAP mixture featuring the use of 45% RAP and a softer binder (PG 58-28).
- 60_HR_L_RA: a BMD-optimized HRAP mixture featuring the use of 60% RAP, a softer binder (PG 58-28), and an RA.

The BMD-optimized mixtures were designed following the VDOT BMD special provisions using Approach D (Performance Only). The volumetric properties and gradations of all mixtures collected during design with various performance testing details can be seen in Table 1. It should be noted that the relatively higher binder content of mixture 45_HR may have made the mixture exhibit performance properties that are not necessarily comparable to the other five mixtures. During the production process, four samples were systematically taken for each mixture, with the second sample being reserved for subsequent advanced-level laboratory performance evaluations. Detailed volumetric characteristics and gradations of all mixtures, as observed in the second sample obtained during production, are presented in Table 2. During production, all evaluated mixtures except mixture 45_HR had binder contents similar to or slightly deviated from the design and within the allowable tolerance according to the VDOT specifications. However, an increase of almost 0.4% in binder content was observed for mixture 45_HR. No major changes in aggregate gradations between design and production were observed for any of the evaluated mixtures, regardless of the RAP content.

### 5.2. Design of APT Test Cells

**Figure 2:**
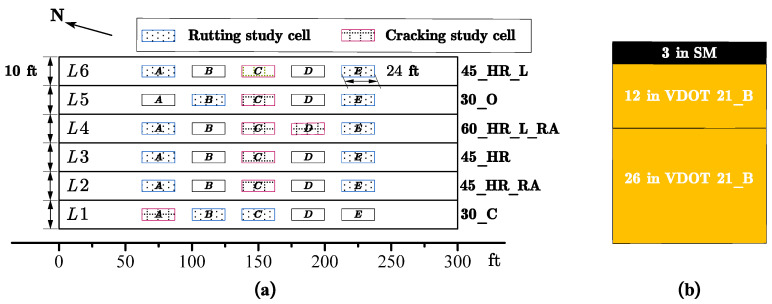
(**a**) The Site Layout for the APT Experiment; (**b**) Pavement Structure.

Six APT lanes were constructed, and each lane was designated to evaluate one of the six mixtures under full-scale accelerated loading. Each lane had a length of 91.4 m (300 ft) and a width of 10 ft. Within each lane, five test cells (labeled A through E) were established. Test cells A, C, and E were specifically designated as the test cells for comprehensive data collection. These test cells were instrumented with a variety of sensors, including strain gauges, pressure cells, moisture sensors, and thermocouples, to collect pavement response data. Test cells B and D served as backup test cells to address unforeseen issues or unexpected damage during the experiment. The test cells were strategically positioned within each lane, spanning the center 61.0 m of 91.4 m (200 ft of the 300 ft) lane; each test cell had a testing length of 7.3 m (24 ft). Space between each test cell provided room for the HVS support structure. Figure 2a provides a visual representation of the site layout for the APT experiment. The pavement structure, shown in Figure 2b, was consistent across all six constructed lanes. Each lane was built with one of the six evaluated mixtures consisting of two 38.1-mm (1.5-inch) lifts, with a total thickness of 76.2 mm (3 inches). Each research mixture was placed on top of a 304.8 mm (12-inch) VDOT 21B base layer, followed by an additional 660.4 mm (26-inch) layer of VDOT 21B as a subgrade layer placed over a reinforced foundation.

### 5.3. Loading Protocols

Two studies were conducted per lane, one for rutting evaluation and another for cracking evaluation. Specific cells were assigned for each study. The key distinguishing factors between the rutting and cracking studies were the variation in testing temperatures, loading protocols, and wandering settings. For this paper, the focus is the investigation of rutting performance within the 12 rutting test cells, two cells in each of the six lanes.

The loading and repetitions were converted into equivalent single axle loads (ESALs), with the target of achieving approximately 500,000 ESALs in each rutting test cell. The number of ESALs was calculated using Equation (1). The 18-kip single-axle equivalency concept was introduced based on the AASHTO road testing completed in 1961 [24]. One pass at the 40 kN (9000 lbf), 53.4 kN (12,000 lbf), and 66.7 kN (15,000 lbf) load levels corresponded to 1.00, 3.35, and 8.55 ESALs, respectively.

(1) $$ESALs={\left(\frac{\mathrm{wheel}\;\mathrm{load}\;\mathrm{in}\;\mathrm{lb}}{9000}\right)}^{4.2}$$

The loading protocol implemented within each test cell followed a specific pattern. Initially, a wheel load of 40 kN (9000 lbf) was applied for the first two weeks, resulting in an estimated accumulation of approximately 45,000 ESALs. Subsequently, the load was increased to 53.4 kN (12,000 lbf), maintained for approximately one additional week, and correspondingly, approximately 200,000 cumulative ESALs were achieved. This was followed by a further increase to 66.7 kN (15,000 lbf), maintained for approximately one more week to attain the desired target ESALs. This progressive loading protocol was designed to expedite the development of pavement distresses. Wandering was set to a maximum of 50.8 mm (2 inch) on either side of the centerline. The internal pavement temperature was maintained at 40 °C (104 °F), measured by a thermocouple embedded at a depth of 50.8 mm (2 inch) beneath the pavement surface. The 40 kN (9000 lbf) load level was selected to simulate half of an 80 kN (18,000 lbf) standard axle load. Throughout the test period, the HVS operated continuously except for regular daily maintenance and occasional repairs. Additionally, temporary staff shortages during the COVID-19 period may have impacted on the testing schedule.

### 5.4. Experiment Timeline

**Figure 3:**
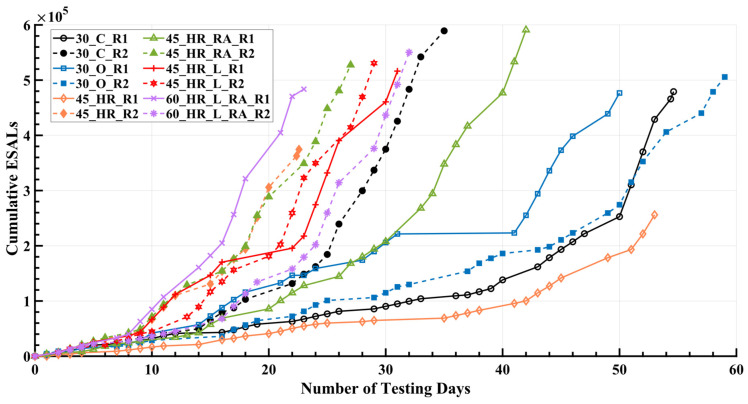
Loading Protocols for Various Test Cells. ESALs = equivalent single axle loads; R1 = rutting Cell 1; R2 = rutting Cell 2.

The APT BMD experiment was conducted over a period of approximately three years. Detailed information regarding the loading timeline and applied loading conditions for the pavement during the testing period can be found in Table 3. The notation R1 was used to indicate rutting Cell 1, which underwent testing prior to rutting Cell 2, which was denoted by R2. This differentiation was made to facilitate the subsequent explanation of potential environmental aging effects. The loading protocols employed for the test cells are summarized in Figure 3. The slope of the curves depicted in the figure indicated the loading rate, which might be influenced to a minor extent by daily management practices, was primarily driven by the magnitude of the applied load. Specifically, as the loading magnitude increased, the slope of the curves became steeper. Besides, it was observed that the accumulated ESALs for cells 45_HR_R1 and 45_HR_R2 were relatively lower than other test cells. This can be attributed to the fact that these cells experienced rutting failure at an earlier stage compared to the remaining cells. It is important to note that, due to the arbitrary arrangement of lanes and material allocations, the test IDs have been redefined in Table 2 for clarity and consistency in subsequent discussions. Initially, the dual tire assembly ran unidirectionally at a constant speed of 6.4 km/h (4 mph). However, due to equipment aging issues, the loading speed was increased to 9.7 km/h (6 mph) on 6 May 2021. Consequently, most of the test cells were subjected to loading at 9.7 km/h (6 mph), while 3 test cells were under loading at 6.4 km/h (4 mph).

### 5.5. Rut Depth Measurement

**Figure 4:**
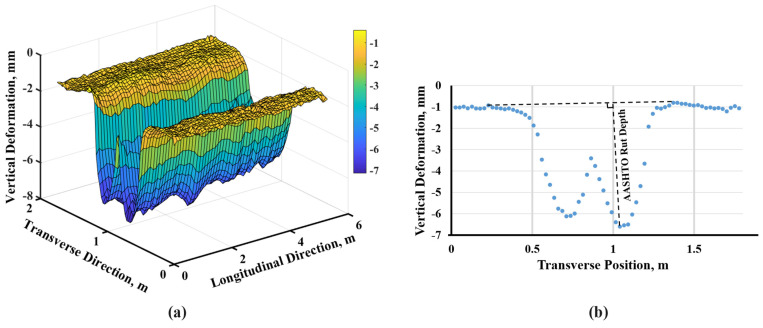
An Example of Measurement in the Experiment for Cell L1B on 11 October 2022: (**a**) 3-D Laser Profile; (**b**) Rut Depth in a Specific Transverse Plane.

The laser profiler, positioned on the carriage of the HVS, as shown in Figure 1b, was used to conduct regular surface scans of the test bed. These scans were performed on a daily or bi-daily basis, excluding weekends and vacation periods. The laser profilers captured surface elevation measurements at 101.6 mm (4-inch) intervals across the longitudinal span of the HVS test cell (7.3 m [24 ft]), resulting in the generation of a row of data containing 69 points. However, due to the location of the laser profiler at the head of the mounted carriage (Figure 1b), only the initial 53 points, equivalent to a distance of 5.4 m (17 ft. 8 in.), were deemed to provide effective measurement data. Subsequent to completing one set of longitudinal measurements, the laser profiler was shifted inward by 25.4 mm (1 inch) in the transverse direction. Within the transverse direction, a total of 70 measurement points were established, distributed symmetrically with 35 points on each side of the wheel path’s center. A daily surface scan, presented as a three-dimensional (3-D) profile consisting of 53 transverse planes, as shown in Figure 4a, effectively visualized the deformation of the pavement surface.

According to the AASHTO glossary [25] and ASTM E 1703/E 1703M, rut depth is defined as “the maximum measured perpendicular distance between the bottom surface of the straightedge and the contact area of the gage with the pavement surface at a specific location” [26]. This concept is visually represented in Figure 4b. In the subsequent analysis, the median value of rut depth within the 3-D profile was selected as a representative indicator of the magnitude of rutting, which was used to analyze and quantify the progression of rutting.

## 6. APT Results and Discussion

**Figure 5:**
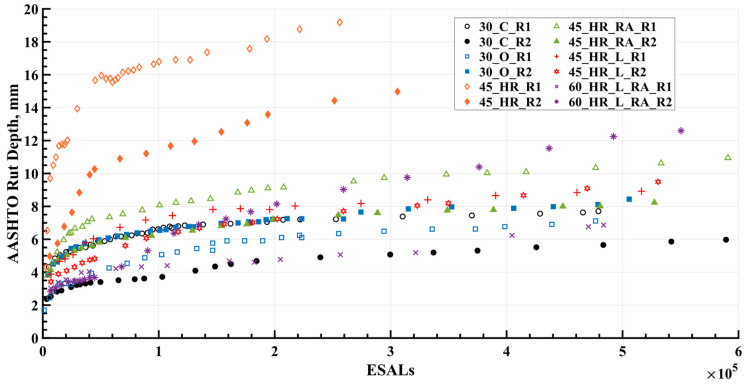
Rutting Depth for 12 Rutting Test Cells. ESALs = equivalent single axle loads; R1 = rutting Cell 1; R2 = rutting Cell 2.

Figure 5 shows the rut depth measurements collected within the 12 rutting test cells. It was evident that mixture 45_HR showed the poorest rutting resistance compared to the other five mixtures, as indicated by its rapid accumulation of rut depth, reaching 12.5 mm, which was considered a potential threshold candidate for rutting failure in pavements [21]. This performance can be attributed to the significantly higher flexibility resulting from the high OBC used in the design and production of mixture 45_HR. On the other hand, the rutting performance of the remaining five mixtures appeared comparable, except for 60_HR_L_RA_R2, which exhibited a significant difference from 60_HR_L_RA_R1 after increasing the load magnitude from 40 kN (9000 lbf) to 53.4 kN (12,000 lbf), with its rut depth approaching 12.5 mm at the end of loading cycles. Nevertheless, despite a thorough examination of the five mixtures, determining their rutting performance ranking proved to be difficult. This challenge arose from the variance in test results observed between the corresponding test cells, R1 and R2, which can confound a simple ranking approach. Consequently, further investigation was warranted to elucidate the underlying factors that contributed to the observed differences in performance between test cells R1 and R2, thereby facilitating a comprehensive evaluation and comparison of the various mixtures.

### 6.1. Environmental Aging Effect

Upon placement and exposure to the environment, asphalt mixtures undergo an aging process characterized by the oxidation of the asphalt binder, leading to changes in the viscous properties of the binder and subsequently impacting the overall properties of the mixture [27]. The measured rut depth results in Figure 5 clearly demonstrate the influence of aging effects, as the later-tested test cells (R2) outperformed their corresponding earlier-tested test cells (R1) in terms of rutting performance, representing mixtures 30_C, mixture 45_HR, mixture 45_HR_RA, and mixture 45_HR_L. This observation aligned with the experiment timeline presented in Table 2, where test cells R2 were tested 448, 536, 269, and 262 days later than their corresponding test cells R1 for mixture 30_C, mixture 45_HR, mixture 45_HR_RA, and mixture 45_HR_L, respectively. Instances of such aging effects were commonly observed in the APT BMD experiment. For instance, in an APT conducted at the National Airport Pavement and Materials Research Center, it was found that the outdoor test sections exhibited superior rutting performance compared to their corresponding indoor sections, attributed to the environmental aging experienced by the outdoor sections [22]. Furthermore, aging effects were considered and investigated in APT testing conducted by the FDOT [28], the Indiana DOT [29], and the University of California Pavement Research Center in Davis [30].

However, it was not only the aging effect but also the construction variability that played a significant role in investigating the resultant rut depth, which was believed to be a major contributing factor for the exception observed in mixture 30_O and mixture 60_HR_L_RA from the aging effect. For mixture 30_O, the test cells 30_O_R1 and 30_O_R2 were tested back-to-back, and considering the relatively small difference in rut depth between them, it can be inferred that the construction variability of 30_O_R1 and 30_O_R2 was within a reasonable range and the influence of aging was minimal. As for mixture 60_HR_L_RA, test cell 60_HR_L_RA_R2 was tested 251 days later than 60_HR_L_RA_R1, and the scenario where the rut depth of 60_HR_L_RA_R1 exceeded that of 60_HR_L_RA_R2 was observed only under the 40 kN (9000 lbf) loading condition. However, as the applied loading magnitude increased to 53.4 kN (12,000 lbf), the rut depth of 60_HR_L_RA_R2 immediately surpassed that of 60_HR_L_RA_R1. Within 500,000 ESALs, 60_HR_L_RA_R2 closely approached the failure threshold of 12.5 mm. It is believed that test cells 60_HR_L_RA_R1 and 60_HR_L_RA_R2 exhibited significant construction variability, surpassing the influence of aging. Notably, in the earlier laboratory performance tests, both the APA test and the SSR test (to be discussed in detail in the following sections) indicated relatively inferior rutting resistance for mixture 60_HR_L_RA compared with the other five mixtures, only slightly better than that of mixture 45_HR. Considering this, the performance of test cell 60_HR_L_RA_R2 aligned more closely with expectations, as it had substantial rut depth at the end of testing life.

### 6.2. Normalization

The aging process significantly impacted the rutting performance of the respective mixtures. Prior to comparing the rutting performance across distinct mixtures and establishing a correlation with results derived from laboratory testing, it was imperative to normalize them to the same set of initial conditions. To further this understanding, pavement age should be integrated as a significant variable within the rutting model. Subsequently, an empirical model expressed in Equation (2) [23] was formulated to simulate the evolution of rut depth for each lane (two test cells).

(2) $$RD=\alpha \cdot ESAL^{\beta_{1}}\cdot Age^{\beta_{2}}$$

where *RD* is the AASHTO rut depth in mm; *Age* is the number of days passed from pavement paving date to the rutting measurement; and α, $\beta_{1}$, $\beta_{2}$ are regression coefficients.

The regression coefficients and results of the statistical analysis using the rutting model were summarized in Table 4. A positive coefficient ($\beta_{1}$ > 0) indicated that rut depth increases with an increase in ESALs. Conversely, a negative coefficient ($\beta_{2}$ < 0) suggested that aging had a negative impact on rut depth, meaning that as the pavement aged, it contributed positively to the resistance against rutting. The high values of $R^{2}$, ranging from 0.95 to 0.99, indicated a strong fit of the proposed rutting model to the measured data. Moreover, the high value of $R_{adjust}^{2}$ highlighted the significance of both ESALs and age as influential factors in determining the rutting depth of the test cells. The combined effect of these two parameters explained a minimum of 94.78% (obtained in mixture 45_HR) of the variation in the measured rutting data during the experiment. Notably, loading speed was extended into Equation (2) to account for its potential influence on rutting performance, particularly for mixtures 30_O and 45_HR. However, the results of the regression and statistical analyses revealed that loading speed was not a significant predictor in these cases. This outcome could be attributed to the controlled nature of loading speed, which was implemented using the half-sine function. The rutting measurements were found to be less sensitive to small variations in loading speed compared to the effects of ESALs and pavement age. Therefore, Equation (2) was deemed sufficient for regression analysis across all test lanes.

**Figure 6:**
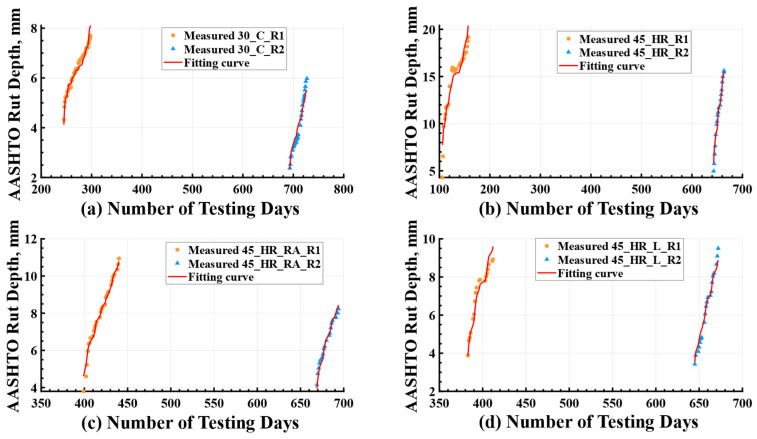
Fitting Result for Testing Lanes of (**a**) Mixture 30_C; (**b**) Mixture 45_HR; (**c**) Mixture 45_HR_RA; (**d**) Mixture 45_HR_L. ESALs = equivalent single axle loads; R1 = rutting Cell 1; R2 = rutting Cell 2.

**Figure 7:**
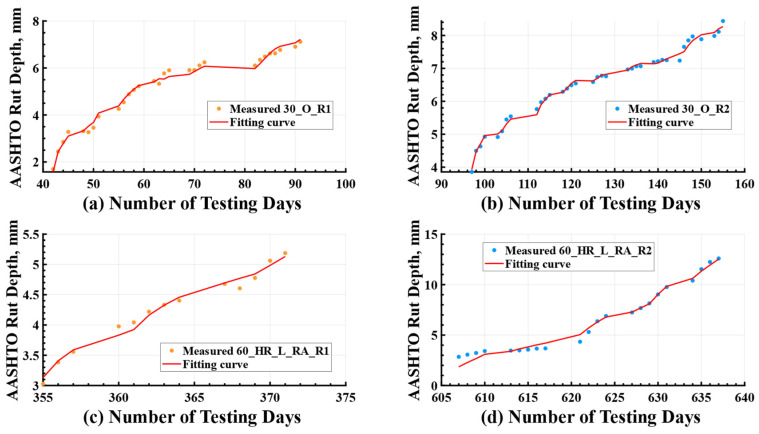
Fitting Result for Testing Cells: (**a**) Cell 30_O_R1; (**b**) Cell 30_O_R2; (**c**) Cell 60_HR_L_RA_R1; (**d**) Cell 60_HR_L_RA_R1. ESALs = equivalent single axle loads; R1 = rutting Cell 1; R2 = rutting Cell 2.

It is noteworthy that the rutting model was intended to capture the measured data for two test cells within a single lane in Table 3. However, as discussed earlier, deviations from the expected behavior were observed in the cases of mixture 30_O and mixture 60_HR_L_RA. For mixture 30_O, the two rutting cells exhibited similar outcomes due to their close testing times, while for mixture 60_HR_L_RA, the influence of construction variability surpassed the effects of aging. Consequently, separate curve-fitting analyses were conducted for their corresponding rutting cells, namely R1 and R2, in these exceptional scenarios. The fitted curves depicting the rutting depth were then juxtaposed against the corresponding experimental measurements, as shown in Figure 6. Owing to the disparities in testing periods (Table 3), the measured rut depth in test cell R2 (blue point) frequently exhibited a smaller magnitude compared to that in test cell R1 (orange point), thereby highlighting the presence of an aging effect. This phenomenon is effectively showcased in Figure 6 through the effective utilization of the rutting model. Additionally, Figure 7 specifically represents the exceptional cases. The visual representations presented in these figures affirmed that the proposed rutting model effectively captured the diverse rutting behaviors exhibited by the different test cells.

**Figure 8:**
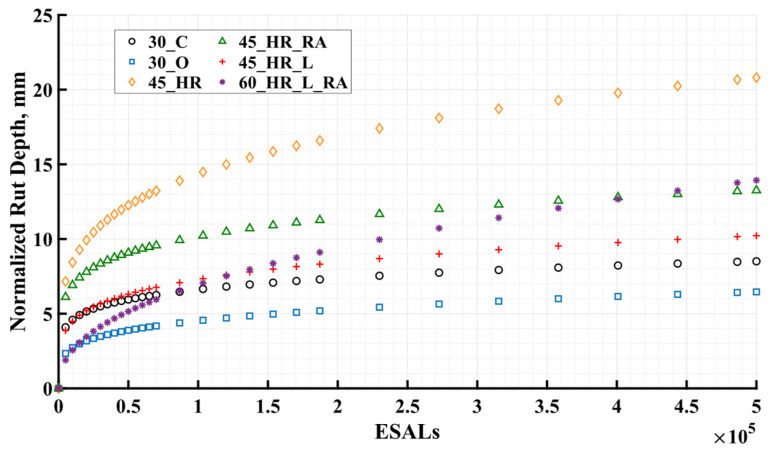
Normalized AASHTO Rut Depth Versus ESALs. ESALs = equivalent single axle loads.

Through the application of regression analysis, the rutting model facilitated the prediction of rutting development for each mixture under a well-defined loading protocol. This methodology ensured the comparability of different mixtures, allowing for more controlled analysis of variables. In addition to normalizing testing periods or the environment aging effect, the rutting model effectively addressed the disparities that arise from the use of APT loading protocols under realistic management conditions (refer to Figure 3). Assuming identical testing start time was applied to all lanes; this followed the same regimen as the control mixture 30_C (245 days after paving). Adhering to the predetermined schedule of loading protocols for all test cells, a load of 40 kN (9000 lbf) was applied for the initial 14 days, with 5000 passes per day. Subsequently, a load of 53.4 kN (12,000 lbf) was applied for 7 days, followed by a final load of 66.7 kN (15,000 lbf) for a duration of one week (7.32 days), resulting in a total of 500,000 ESALs. The entire loading process spanned a duration of 28.32 days. The progression of rutting depth for each lane is shown in Figure 8.

Upon visual inspection, the normalized results demonstrated comparability with the measured results (refer to Figure 5) among different mixtures, attesting to the consistency between the measured data and the normalization approach. Test cell 30_O_R1 and test cell 30_O_R2 demonstrated remarkably similar rutting performance, supporting the use of their average value in Figure 8 for subsequent comparisons with other mixtures. Conversely, test cell 60_HR_L_RA_R2 displayed augmented sensitivity to increasing load magnitude when contrasted with test cell 60_HR_L_RA_R1. Drawing upon the insights garnered from the laboratory rutting performance tests conducted on mixture 60_HR_L_RA at varying complexity levels, the rut depth development of test cell 60_HR_L_RA_R2 was deemed representative and employed for subsequent comparisons with different mixtures in Figure 8. In terms of rutting performance, mixture 45_HR exhibited the highest rut depths, followed by mixtures 60_HR_L_RA and 45_HR_RA, and then mixtures 45_HR_L, 30_C, and 30_O. It was observed that all BMD HRAP mixtures showed higher rut depths compared to the control mixture 30_C. This can be attributed to the inclusion of a high binder content in BMD HRAP mixtures to enhance cracking resistance, which may have compromised rutting resistance. It is worth noting that, despite the similarity in rutting performance between mixture 30_O and mixture 30_C (Figure 5), the testing for cells containing mixture 30_O was conducted 203 days earlier than that for mixture 30_C. Considering the aging effect, after normalization, mixture 30_O exhibited better rutting performance compared to mixture 30_C. Furthermore, at the end of 500,000 ESALs, certain mixtures, namely 45_HR, 45_HR_RA, and 60_HR_L_RA, exceeded the example failure limitation of 12.5 mm in terms of rut depths. It should be noted that the rutting failure threshold in APT may require further consideration due to the accelerated manners of APT, which involves incremental loading protocols and high testing temperatures.

## 7. Correlation between APT Measurements and Laboratory Test Results

Two performance tests were used to evaluate the rutting resistance of the six mixtures. These tests encompassed two levels of testing: intermediate (APA rut test) and advanced (SSR test). Subsequent sections primarily focused on establishing and assessing the correlation between normalized APT rut depth and laboratory rutting performance tests.

### 7.1. APA Rut Test

The APA rut test was performed on specimens prepared from loose mixtures obtained during production in accordance with AASHTO T 340 [25]. The test specimens had a diameter of 150 mm and a height of 75 ± 2 mm. The specimens were compacted using a Superpave gyratory compactor to achieve an air void content of 7 ± 0.5%. This laboratory test aimed to simulate rutting by subjecting a loaded wheel in cyclic motion over a pressurized rubber tube positioned along the surface of the specimens. The test was performed at a temperature of 64 °C, which corresponds to the high-temperature PG of asphalt binders typically used in Virginia (i.e., PG 64S-22). The deformation of the specimen, measured after applying 8000 loading cycles, is referred to as the APA rut depth. Two replicate tests (four specimens in total) were conducted for each mixture, and the average results were reported. In 2017, an initial effort was undertaken by researchers at the VTRC to provide benchmark indications of performance for a number of asphalt mixtures produced and sampled in 2015 [11]. The evaluation was conducted on specimens fabricated from reheated plant-produced mixtures. Numerous performance tests were considered in the laboratory to evaluate the selected mixtures. Among these tests, the APA was selected to assess the resistance to rutting. An initial threshold of 8 mm after 8000 loading cycles at 64 °C was then developed, verified, and validated [10,11].

In this current study, the APA rut test was performed on two types of specimens in this study:

- Non-Reheats: laboratory-compacted non-reheated specimens that were compacted on-site by the producer staff without reheating the loose mixture sampled at the plant and tested in the research team’s laboratories by their staff.
- Reheats: laboratory-compacted reheated specimens that were compacted and tested in the research team’s laboratories by their staff after reheating the loose mixture sampled at the plant. Mixtures were not subjected to any additional oven aging.

The testing involved four sets of non-reheated specimens and four sets of reheated specimens procured at distinct intervals during plant production. It aimed to assess the impact of reheating the mixtures on the APA rut test results as well as the remaining VDOT BMD performance tests (Cantabro and IDT-CT). The data relevant to this assessment was only presented within the objectives of this paper.

### 7.2. SSR Test

The SSR test, conducted in accordance with AASHTO TP 134 [25], was employed to evaluate the rutting resistance of mixtures under repetitive loading, simulating the traffic-induced conditions experienced by pavements. Four specimens were subjected to confined cyclic compression testing at two different temperatures, $T_{H}$ (55 °C) and $T_{L}$ (26 °C). In both cases, the specimens were subjected to three sets of 200-cycle loading blocks, each at three deviatoric stress levels, while maintaining a constant confining pressure of 69 kPa. During the testing, the permanent deformation of specimens was measured along with their deviatoric stress, loading history, and temperature. The rutting strain index (RSI), a metric adopted by the Federal Highway Administration (FHWA) and supported by the SSR shift model, served as an indicator of rutting. The RSI represents the average permanent strain, expressed as a percentage, resulting from the repetition of 30 million ESALs over a period of 20 years on a standard pavement structure [31]. A lower RSI value indicates a greater resistance to rutting for a given mixture. It is noteworthy that only the second set of reheated mixtures sampled from plant production, compacted in the laboratory, underwent this advanced-level rutting performance evaluation.

### 7.3. Correlation

The Pearson correlation coefficient was used to examine the strength and direction of the linear relationship between APT results and laboratory rutting performance tests (APA and SSR tests). The results are summarized in Table 5. Among these, the APT results were selected based on the normalized AASHTO rut depth for each lane at the culmination of 500,000 ESALs, as shown in Figure 8. For mixture 30_O, the average value of 6.46 mm was obtained by considering the mean rut depth measurements derived from test cell 30_O_R1 (6.25 mm) and test cell 30_O_R2 (6.67 mm). Conversely, for mixture 60_HR_L_RA, the rut depth of 13.93 mm at the termination of 500,000 ESALs was recorded from test cell 60_HR_L_RA_R2 and used for subsequent analysis. In the study, the SSR test results derived from the second set of reheated mixtures were employed as the representative advanced level indicator for rutting performance, as displayed in Table 5. Correspondingly, the APA test results from this identical set of reheated mixtures were also represented. Finally, the average values computed from four sets of non-reheated specimens and four sets of reheated specimens, alongside their standard deviations, were included in Table 5 for comprehensive comparison.

**Figure 9:**
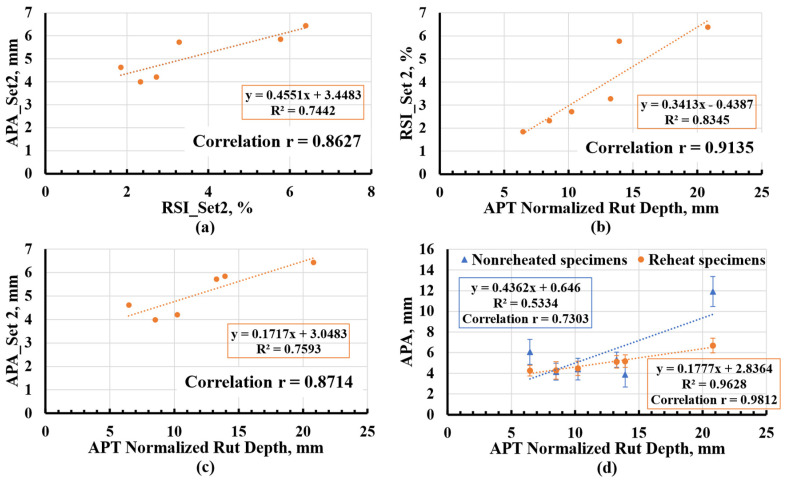
Rutting Testing Results Plot: (**a**) APA vs. RSI; (**b**) APA vs. APT Normalized Rut Depth; (**c**) RSI vs. APT Normalized Rut Depth; (**d**) Correlation Analyses. APT = Accelerated Pavement Testing; APA = asphalt pavement analyzer; RSI = rutting strain index.

The rutting testing results, obtained from pairwise comparisons between APA_Set2 and RSI_Set2, between RSI_Set2 and APT normalized rut depth, and between APA_Set2 and APT normalized rut depth are presented in Figure 9a–c, respectively. Figure 9d combines the comparison between the APA average value of four sets of non-reheated specimens and APT normalized rut depth and between the APA average value of 4 sets of reheated specimens and APT normalized rut depth. The error bar indicates their corresponding ±1 standard deviation. The use of linear regression curves with its R-square provided a characterization of the relationships between any two testing methods. Furthermore, the assessment of correlation was quantified through the use of correlation coefficients (r values). The r values of correlations between APA_Set2 and RSI_Set2, RSI_Set2 and APT normalized rut depth, and APA_Set2 and APT normalized rut depth were 0.863, 0.914, and 0.871, respectively. These elevated r values indicated a significant positive correlation among the rutting performance tests considered in this study.

The highest positive correlation (r = 0.9812) was observed between the APA average value of four sets of reheated specimens and the APT normalized rut depth. This aligned with the anticipated outcome, as averages derived from samples collected at various times during production or APT construction provided a more representative measure. Conversely, the weakest correlation (r = 0.73) was seen between the APA average value of four sets of non-reheated specimens and the APT normalized rut depth. The inconsistency in results can primarily be attributed to production variability and contractor-induced variability when rushing to prepare non-reheated specimens at the right air void during production. For example, this resulted in significant differences between the non-reheated and reheated sets of mixtures 30_O and 45_HR. Basically, the non-reheated specimens from mixtures 30_O and 45_HR had considerably more air voids than their reheated and designed counterparts, leading to greater APA rut depth.

Assuming the influence of production is discounted, the rutting performance of non-reheated sets could be comparable to that of the reheated sets. Consequently, a stronger correlation between the performance of non-reheated specimens and the APT test results could possibly be extrapolated. Moreover, a linear relationship aptly described the connection between different test results, particularly evident in the R-square value of 0.9628 for the APA average value of four sets of reheated specimens and the APT normalized rut depth, highlighting a robust linear relationship between APA and APT normalized rut depth.

Aside from examining the correlation between laboratory-scale and full-scale testing, another crucial consideration of this APT experiment was the validation of the VDOT rutting threshold of 8 mm in terms of APA rut depth (determined on reheated specimens). For instance, the FHWA recommended an RSI value ranging between 4% to 12% for standard traffic (ESALs < 10 million) [31]. The corresponding APA rut depth, determined using the linear regression shown in Figure 9a, ranged between 5.269 mm and 8.910 mm, with an average of 7.090 mm. Likewise, for the recommended RSI of 4% to 12%, the corresponding normalized APT rut depth ranged between 13.005 mm and 36.445 mm, as derived from Figure 9b. By using the regression analysis curve for reheated sets, the corresponding APA rut depth ranged between 5.15 mm and 9.31 mm, with an average value of 7.23 mm. This value was lower than the VDOT current APA rut depth threshold of 8 mm. It should be noted that the allowable HVS rut depth of 12.5 mm, determined according to the literature, would lead to an APA rut depth of 5.06 mm, which is significantly lower than the 8 mm VDOT BMD threshold. From another viewpoint, considering the rutting failure limitation of 24.5 mm allowed in airfield pavement [22], the corresponding rut depth was observed to be 7.19 mm from Figure 9d. All of that indicates that the VDOT may need to revise its BMD specification by reducing the APA rut depth threshold to at least 7.000 mm. However, it is vital to recognize that these are initial findings that are constrained by the limited number and types of mixtures evaluated in this study. Furthermore, these observations are pending further substantiation from real-world field performance data of sections constructed in Virginia.

## 8. Summary of Findings, Conclusions, and Future Work

This research provided a comprehensive evaluation of the permanent deformation behavior of a control mixture and five BMD-optimized SMs through full-scale testing at an APT facility under incremental loading conditions. The incorporation of various combinations of RAP contents, binder PGs, RA, and WMA additive, including four HRAP mixtures, allowed for a thorough investigation of their rutting performance. This paper specifically documented the design of the APT BMD experiment, the traffic test plan, and the measurement of rutting performance.

The empirical rutting model proposed in this study effectively accounted for the observed environment aging effect and facilitated the normalization of measured rut depth. The results demonstrated that all BMD HRAP mixtures exhibited higher rut depths compared to the control mixture 30_C. This can be attributed to the higher binder content in BMD HRAP mixtures, which aimed to enhance cracking resistance but compromised rutting resistance. Notably, mixture 45_HR showed a high likelihood of reaching the rutting failure even under a loading of 40 kN (9000 lbf). Nevertheless, when considering the aging effect, the BMD-optimized mixture 30_O displayed better rutting performance compared to the control mixture.

Correlation analysis revealed significant and strong positive correlations between APT normalized rut depth and multi-level laboratory rutting performance tests. Remarkably, the highest positive correlation (r = 0.9812) was observed between the APA average value of reheated specimens and the APT test.

Finally, this research provided a steppingstone towards the validation of the VDOT BMD APA rut depth threshold of 8 mm based on actual full-scale accelerated testing and recommended RSI value for standard traffic. The proposition to lower the current limit from 8 mm to 7 mm is advised as a means to enhance the performance of asphalt mixtures from a rutting point of view.

Subsequent research will explore the impact of incremental loading protocols on the mixtures using the time-hardening approach and will assess the potential applicability of the conventional load equivalency coefficient of 4.2 to BMD mixtures in general and HRAP mixtures in particular. Concurrently, an in-depth study on the cracking section is in progress, with an attempt to validate the VDOT BMD threshold of 7.5% and 70 for mass loss and CT index by means of Cantabro tests and IDT-CT, respectively.

## Figures and Tables

**Table 1:** Volumetric Properties and Gradations for HVS Mixtures—Design.

Mixture ID	Mixture I 30_C	Mixture II 30_O	Mixture III 45_HR	Mixture IV 45_HR_RA	Mixture V 45_HR_L	Mixture VI 60_HR_L_RA
**Description**	Non-BMD	BMD	BMD HRAP	BMD HRAP	BMD HRAP	BMD HRAP
**Composition**						
RAP Content, %	30	30	45	45	45	60
Asphalt Binder	PG 64S-22	PG 64S-22	PG 64S-22	PG 64S-22	PG 58-28	PG 58-28
Additives	WMA	WMA	WMA	WMA + RA	WMA	WMA + RA
**Property**						
Ndesign, gyrations	50	50	50	50	50	50
NMAS, mm	9.5	9.5	9.5	9.5	9.5	9.5
Asphalt Content, %	5.6	6.0	6.8	6.2	6.0	6.0
Rice SG (Gmm)	2.531	2.517	2.497	2.519	2.521	2.538
VTM, %	4.0	2.1	5.2	2.7	4.2	1.5
VMA, %	16.8	15.9	20.2	16.9	17.9	15.3
VFA, %	76.2	87.0	74.3	84.0	76.6	90.0
FA Ratio	1.0	1.1	1.2	1.2	1.3	1.5
Mixture Bulk SG (Gmb)	2.429	2.465	2.367	2.452	2.415	2.500
**Performance Properties at Optimum Asphalt Binder Content**						
Cantabro Mass Loss, %	2.9	3.2	4.1	2.8	2.7	4.0
APA Rut Depth at 64 °C, mm	5.4	4.2	5.6	7.2	4.9	3.7
CT index at 25 °C	58	112	299	96	141	128
**Gradation/Sieve Size**	**% Passing**					
¾ in (19.0 mm)	100.0	100.0	100.0	100.0	100.0	100.0
½ in (12.5 mm)	100.0	100.0	100.0	100.0	100.0	100.0
^3^⁄_8_ in (9.5 mm)	94.0	96.0	93.0	93.0	92.0	92.0
No. 4 (4.75 mm)	66.0	66.0	63.0	63.0	63.0	62.0
No. 8 (2.36 mm)	39.0	41.0	38.0	38.0	40.0	39.0
No. 16 (1.18 mm)	--	--	--	--	--	--
No. 30 (600 µm)	23.0	17.0	18.0	18.0	19.0	20.0
No. 50 (300 µm)	--	--	--	--	--	--
No. 100 (150 µm)	--	--	--	--	--	--
No. 200 (75 µm)	5.8	6.6	7.4	7.4	7.5	8.3

S = standard traffic; BMD = Balanced Mix Design; HRAP = high reclaimed asphalt pavement content mixture; RAP = reclaimed asphalt pavement; WMA = warm mix additives; RA = recycling agent; NMAS = nominal maximum aggregate size; SG = specific gravity; VTM voids in total mixture; VMA = voids in mineral aggregate; VFA = voids filled with asphalt; FA = fines to aggregate; APA = asphalt pavement analyzer; CT = cracking tolerance.

**Table 2:** Volumetric Properties and Gradations for APT Mixtures During Production.

Mixture ID	30_C	30_O	45_HR	45_HR_RA	45_HR_L	60_HR_L_RA
**Description**	Non-BMD	BMD	BMD HRAP	BMD HRAP	BMD HRAP	BMD HRAP
**Composition**						
RAP Content, %	30	30	45	45	45	60
Asphalt Binder	PG64S-22	PG64S-22	PG64S-22	PG64S-22	PG58-28	PG58-28
Additives	WMA	WMA	WMA	WMA + RA	WMA	WMA + RA
**Property**						
N_design_, gyrations	50	50	50	50	50	50
NMAS, mm	9.5	9.5	9.5	9.5	9.5	9.5
Asphalt Content, %	5.44	6.14	7.22	6.15	6.00	6.14
Rice SG (Gmm)	2.604	2.515	2.505	2.546	2.544	2.548
VTM, %	6.0	4.6	0.6	2.6	2.4	1.7
VMA, %	18.6	18.4	17.5	16.8	16.5	15.8
VFA, %	67.5	75.2	96.5	84.6	85.7	89.5
FA Ratio	1.12	1.07	1.21	1.39	1.21	1.35
Mixture Bulk SG (Gmb)	2.448	2.400	2.490	2.480	2.482	2.506
Aggregate Effective SG (Gse)	2.855	2.777	2.820	2.817	2.807	2.820
Aggregate Bulk SG (Gsb)	2.841	2.761	2.799	2.797	2.795	2.794
Absorbed Asphalt Content (Pba), %	0.18	0.21	0.27	0.26	0.16	0.34
Effective Asphalt Content (Pbe), %	5.27	5.94	6.97	5.90	5.85	5.82
Effective Film Thickness (Fbe), µm	9.2	10.9	10.8	9.1	9.7	8.7
E&R Asphalt Binder	76.9–18.3	77.8–20.6	70.8–21.4	76.9–21.3	79.3–18.8	76.4–20.6
**Gradation/Sieve Size**	**% Passing**					
¾ in (19.0 mm)	100.0	100.0	100.0	100.0	100.0	100.0
½ in (12.5 mm)	99.3	99.5	98.0	98.5	98.7	98.4
^3^⁄_8_ in (9.5 mm)	93.7	94.3	91.9	94.2	93.8	93.4
No. 4 (4.75 mm)	64.5	65.4	61.1	66.8	63.3	66.5
No. 8 (2.36 mm)	39.9	41.1	39.0	42.4	41.9	43.6
No. 16 (1.18 mm)	29.4	25.4	25.7	27.0	27.2	29.9
No. 30 (600 µm)	23.5	16.6	18.4	18.8	19.1	21.8
No. 50 (300 µm)	13.9	11.2	13.6	13.4	13.0	15.2
No. 100 (150 µm)	8.2	8.2	10.6	10.2	9.2	10.5
No. 200 (75 µm)	5.9	6.4	8.4	8.2	7.1	7.9

PG = performance grade; S = standard traffic; BMD = Balanced Mix Design; HRAP = high reclaimed asphalt pavement content mixture; RAP = reclaimed asphalt pavement; WMA = warm mix additives; RA = recycling agent; NMAS = nominal maximum aggregate size; SG = specific gravity; VTM voids in total mixture; VMA = voids in mineral aggregate; VFA = voids filled with asphalt; FA = fines to aggregate; E&R = extracted and recovered.

**Table 3:** Summary of Rutting Testing Cells and Timeline Within the APT BMD Experiment.

Test ID	Cell	Paving Date	Test Period		Loading		
			Start	End	Speed	# of Passes	# of ESALs
**30_C_R1**	L1C	20 October 2020	22 June 2021	13 August 2021	9.7 km/h	182,605	479,170
**30_C_R2**	L1B	20 October 2020	13 September 2022	17 October 2022	9.7 km/h	136,422	589,261
**30_O_R1**	L5B	29 April 2020	10 June 2020	30 July 2020	6.4 km/h	116,199	476,737
**30_O_R2**	L5E	29 April 2020	3 August 2020	2 October 2020	6.4 km/h	134,378	505,751
**45_HR_R1**	L3E	15 July 2020	29 October 2020	19 December 2020	6.4 km/h	135,411	256,075
**45_HR_R2**	L3A	15 July 2020	18 April 2022	9 May 2022	9.7 km/h	110,664	374,457
**45_HR_RA_R1**	L2E	17 July 2020	20 August 2021	30 September 2021	9.7 km/h	136,912	590,851
**45_HR_RA_R2**	L2A	17 July 2020	16 May 2022	11 June 2022	9.7 km/h	132,439	527,431
**45_HR_L_R1**	L6E	22 October 2020	8 November 2021	8 December 2021	9.7 km/h	130,645	516,337
**45_HR_L_R2**	L6A	22 October 2020	28 July 2022	25 August 2022	9.7 km/h	130,358	530,659
**60_HR_L_RA_R1**	L4E	23 October 2020	13 October 2021	3 November 2021	9.7 km/h	122,395	483,630
**60_HR_L_RA_R2**	L4A	23 October 2020	21 June 2022	22 July 2022	9.7 km/h	132,661	550,435

APT = accelerated pavement testing; BMD = balanced mix design; R1 = rutting Cell 1; R2 = rutting Cell 2; ESALs = equivalent single axle loads; # of Passes = the number of Passes; # of ESALs = the number of ESALs.

**Table 4:** Summary of the Regression Coefficients and Results of the Statistical Analysis.

	α	$\beta_{1}$	$\beta_{2}$	$R^{2}$	$R_{adjust}^{2}$
**30_C**	13.171	0.170	−0.475	0.9791	0.9784
**30_O_R1**	0.410	0.269	−0.143	0.9900	0.9892
**30_O_R2**	4.256	0.194	−0.375	0.9934	0.9930
**45_HR**	3.810	0.237	−0.253	0.9500	0.9478
**45_HR_RA**	20.229	0.179	−0.495	0.9851	0.9845
**45_HR_L**	1.578	0.215	−0.169	0.9661	0.9641
**60_HR_L_RA_R1**	2.203	0.130	−0.137	0.9810	0.9772
**60_HR_L_RA_R2**	0.120	0.437	−0.175	0.9851	0.9835

HR = high RAP (reclaimed asphalt pavement); C = control; O = optimized; Ri = rutting cell i; RA = recycling agent; L = lower binder grade.

**Table 5:** Rutting Testing Results.

Rutting Performance	Mixture ID					
	30_C	30_O	45_HR	45_HR_RA	45_HR_L	60_HR_L_RA
Normalized APT Rut Depth, mm	8.507	6.460	20.805	13.256	10.221	13.931
SSR RSI (for Set 2), %	2.33	1.85	6.39	3.28	2.72	5.77
APA Rut Depth (for Set 2), %	3.997	4.627	6.445	5.727	4.207	5.855
Average APA Rut Depth for Non-Reheats, mm	4.164	6.076	11.926	5.320	4.414	3.896
Stdev APA Rut Depth for Non-Reheats, mm	0.825	1.222	1.456	0.741	1.055	1.215
Average APA Rut Depth for Reheats, mm	4.288	4.262	6.689	5.117	4.484	5.180
Stdev APA Rut Depth for Reheats, mm	0.838	0.504	0.712	0.598	0.699	0.607

HVS = heavy vehicle simulator; SSR = stress sweep rutting; RSI = rutting strain index; APA = asphalt pavement analyzer; Stdev = standard deviation.

## Data Availability

Raw data were generated at VTTI/VTRC. Derived data supporting the findings of this study are available from the corresponding author [B.T.] on request.
